# Inhibition of *Candida albicans* morphogenesis by chitinase from *Lactobacillus rhamnosus* GG

**DOI:** 10.1038/s41598-019-39625-0

**Published:** 2019-02-27

**Authors:** Camille Nina Allonsius, Dieter Vandenheuvel, Eline F. M. Oerlemans, Mariya I. Petrova, Gilbert G. G. Donders, Paul Cos, Peter Delputte, Sarah Lebeer

**Affiliations:** 1 University of Antwerp, Department of Bioscience Engineering, Research Group Environmental Ecology and Applied Microbiology, Antwerp, Belgium; 2 Department of Obstetrics and Gynaecology, Antwerp University Hospital, Antwerp, Belgium; 3 Femicare Clinical Research for Women, Tienen, Belgium; 4 University of Antwerp, Department of Biomedical Sciences, Laboratory of Microbiology, Parasitology and Hygiene, Wilrijk, Belgium

## Abstract

Lactobacilli have been evaluated as probiotics against *Candida* infections in several clinical trials, but with variable results. Predicting and understanding the clinical efficacy of *Lactobacillus* strains is hampered by an overall lack of insights into their modes of action. In this study, we aimed to unravel molecular mechanisms underlying the inhibitory effects of lactobacilli on hyphal morphogenesis, which is a crucial step in *C. albicans* virulence. Based on a screening of different *Lactobacillus* strains, we found that the closely related taxa *L. rhamnosus*, *L. casei* and *L. paracasei* showed stronger activity against *Candida* hyphae formation compared to other *Lactobacillus* species tested. By exploring the activity of purified compounds and mutants of the model strain *L. rhamnosus* GG, the major peptidoglycan hydrolase Msp1, conserved in the three closely related taxa, was identified as a key effector molecule. We could show that this activity of Msp1 was due to its ability to break down chitin, the main polymer in the hyphal cell wall of *C. albicans*. This identification of a *Lactobacillus-*specific protein with chitinase activity having anti-hyphal activity will assist in better strain selection and improved application in future clinical trials for *Lactobacillus*-based *Candida*-management strategies.

## Introduction

*Candida albicans* is one of the most prevalent fungal pathogens, causing both superficial mucosal candidosis and life-threatening invasive infections^1^. Under normal circumstances, *C. albicans* lives as a commensal on human mucosal surfaces, but can shift to a pathogenic lifestyle after fungal adhesion and overgrowth, followed by tissue invasion and mucosal infection^2^. This process is enabled by hyphal morphogenesis, which implies the reversible transition between unicellular yeast cells and the filamentous hyphal growth form. The hyphal cell wall is more rigid due to higher levels of chitin and is decorated with other (glyco)proteins compared to the cell wall of unhyphenized yeast cells^3^. These characteristics enable the hyphae to penetrate epithelial tissues, damage endothelial cells and provoke an inflammatory response, making hyphal morphogenesis crucial for the virulence of *C. albicans*^4–8^.

*Candida* infections are traditionally treated with antifungal compounds such as azoles, but resistance to azoles is rising and worrisome^9^. In recent years, the concept of targeting virulence factors instead of pathogen viability has become increasingly popular^10^. The shift of *C. albicans* to hyphal growth forms is a prime example of such a virulence process to target. This shift has been linked to disturbances in the human microbiota and a decreased ability of the commensal microbiota to control *Candida* infections^11^. Due to this key role of the commensal microbiota, the potential of probiotics such as lactobacilli to remodel the composition and/or activity of the microbiota is increasingly explored for application in the vaginal tract^12–18^, the oral cavity of elderly^19–21^, and the gastro-intestinal tract of preterm neonates and children^22–25^. However, clinical trials that assess such interventions have not shown a uniform efficacy of the probiotic *Lactobacillus* strains applied. In addition, it was reported that some *Lactobacillus* taxa still occur in high numbers in women suffering from vulvovaginal candidosis^17^. To better understand the molecular basis of the efficacy of *Lactobacillus* strains against *C. albicans*, it is important to identify the probiotic *Lactobacillus* factors that are able to inhibit *Candida* virulence.

Up to now, mechanistic investigations into the anti-*C. albicans* activity of lactobacilli have mainly focused on their *in vitro* growth-inhibitory capacity, which have generally revealed antimicrobial molecules present in the supernatant, including lactic acid and H_2_O_2_^26–32^. These molecules are however produced widespread by lactobacilli and thus cannot explain differences between *Lactobacillus* taxa and strains. A few recent studies also described *Lactobacillus* strains that could interfere with hyphal formation, but effector components remain unidentified^33–35^. Recently, mechanistic research on probiotics has – at least partly – shifted from strain-specific properties to effector molecules that are more conserved over whole taxa^36,37^, since such core effector molecules have broader application potential for probiotic screening and mechanistic understanding. In this study, we aimed to identify anti-*C. albicans* hyphae factors of *Lactobacillus*, by first performing a thorough screening of various strains followed by detailed biochemical analysis of the active molecules.

## Results

### Selected *Lactobacillus* strains show strong hyphae-inhibitory activity

**Figure 1:**
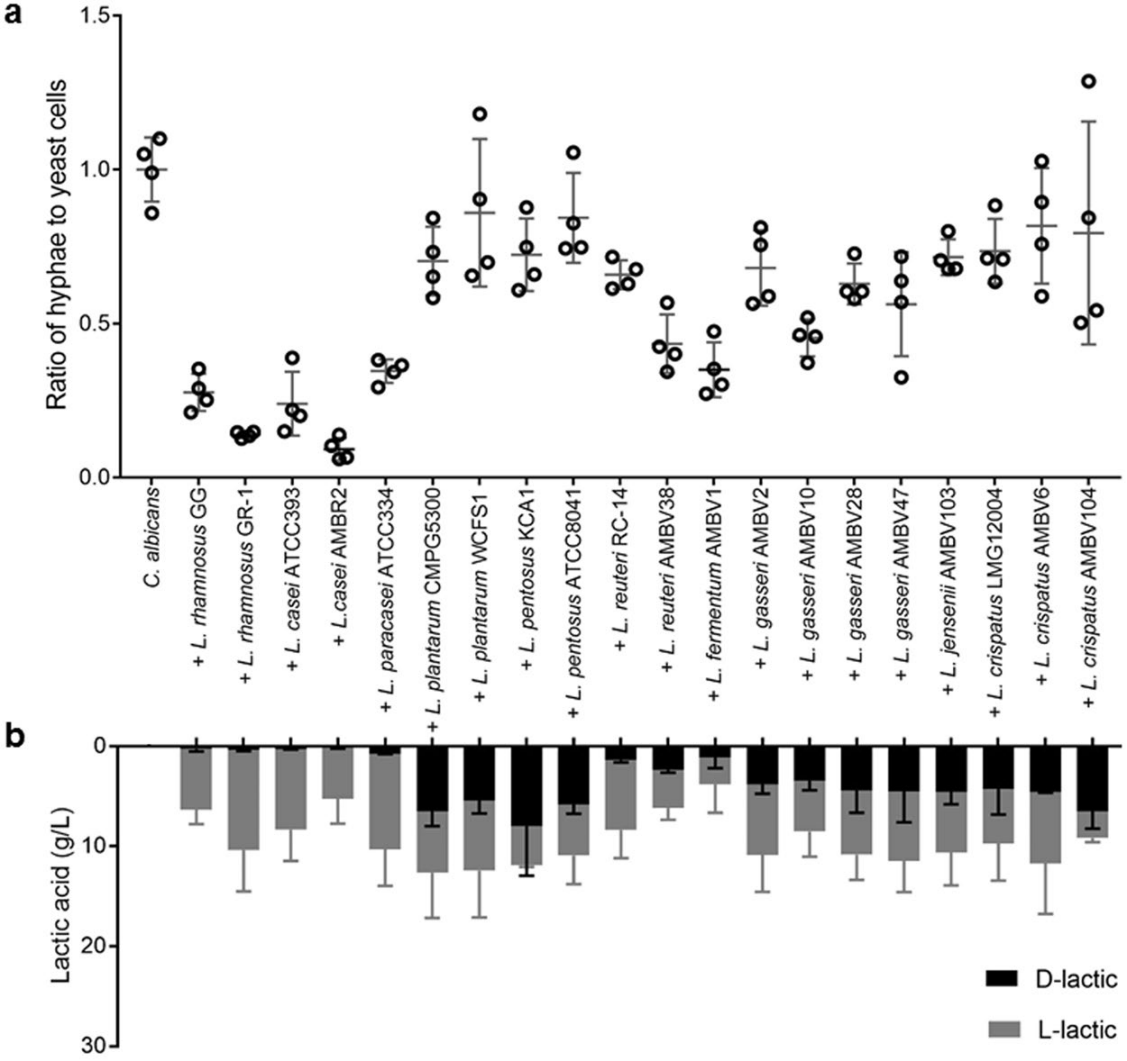
Antihyphal activity and lactic acid production of specific *Lactobacillus* strains. (**a**) Hyphal induction of *C. albicans* (10^6^ cells/ml) during co-incubation with live *Lactobacillus* cells (10^8^ CFU/ml) and (**b**) D- and L-lactic acid production of the investigated *Lactobacillus* strains after growth into stationary phase. The results on hyphal inhibition were normalized to hyphal formation of *C. albicans* solely.

First, we aimed to compare the anti-*Candida* activity between different *Lactobacillus* taxa. Since hyphal morphogenesis is the most important virulence factor of *C. albicans*^6^, we focused on the effect of lactobacilli on serum-induced hyphal morphogenesis. We selected twenty strains available in-house or in the Belgian Co-ordinated Collections of Micro-organisms, representing the different taxa/phylogenetic groups that have been recently described as being mainly nomadic or vertebrate-adapted^38^. Strains were thus selected from the *L. casei* group, *L. plantarum* group, *L. reuteri, L. fermentum, L. gasseri*, *L. jensenii* and *L. crispatus*. The inhibition rates showed large variation among the tested strains, ranging from 91% (*L. casei* AMBR2) to 14% (*L. plantarum* WCFS1) (Fig. 1a).

Lactic acid has been described as key bioactive metabolite of *Lactobacillus*, and is also reported to affect *C. albicans*^39,40^. Therefore we next measured the concentration of D-lactic acid and L-lactic acid in the supernatant of these strains, after growth into stationary phase. All strains were able to produce lactic acid from glucose, although in different ratios of D- and L-lactic acid (Fig. 1b). The level of inhibitory activity of the tested lactobacilli did not increase with an increasing concentration of either isomer, in fact, the inhibitory activity actually showed a negative correlation with the concentration of D-lactic acid (based on Pearson correlation, p-value < 0.0001 for D-lactic acid).

The five best performing strains in our tests all belonged to the *L. casei* group (*L. rhamnosus*, *L. casei* and *L. paracasei*, based on a comparative genome analysis-defined taxonomy as proposed in^41^), suggesting an effector molecule that is shared among these taxa.

### The major peptidoglycan hydrolase of *L. rhamnosus* GG and lactic acid jointly mediate *C. albicans* hyphae inhibition

**Figure 2:**
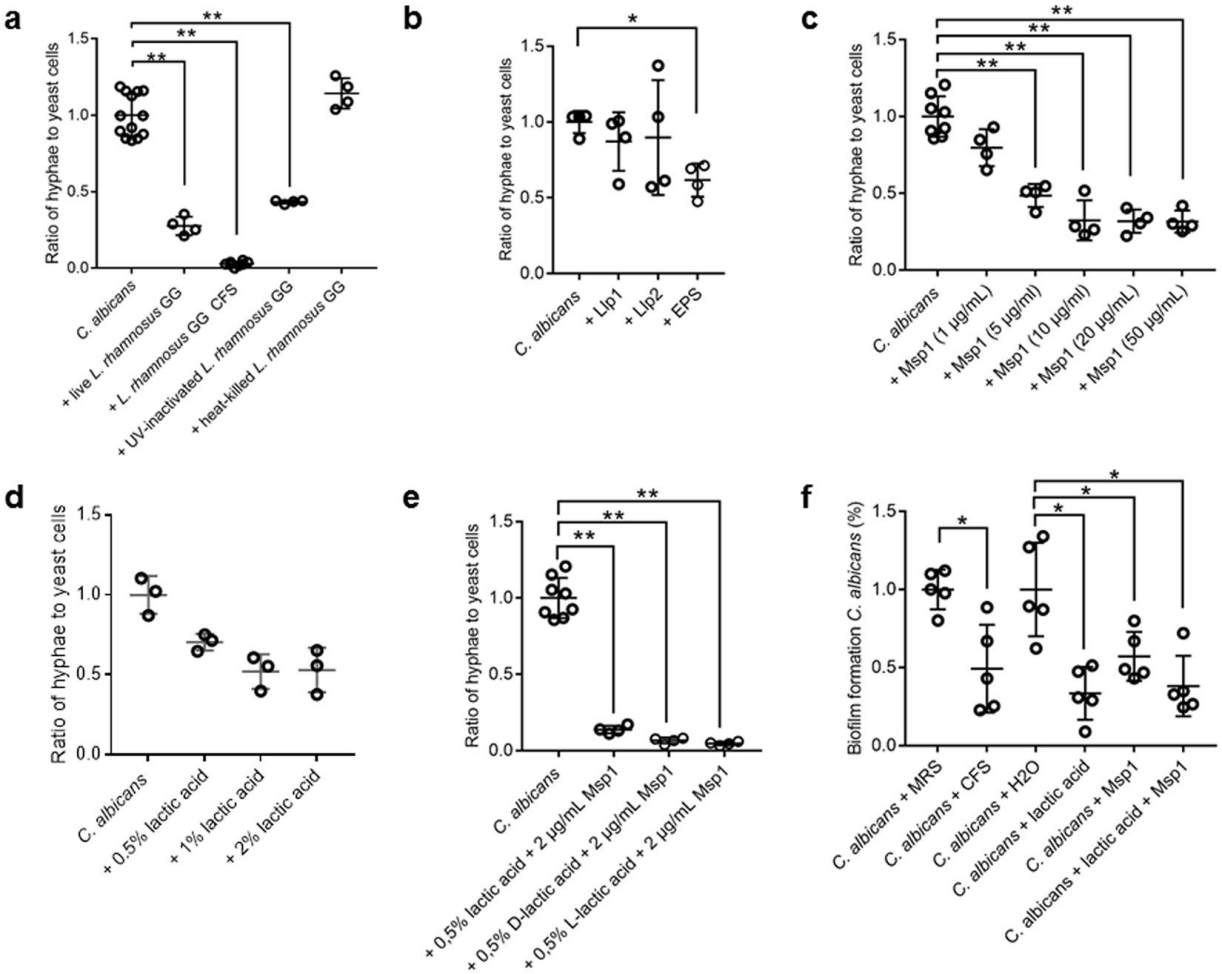
Inhibition of *C. albicans* hyphae by *L. rhamnosus* GG and its components. Hyphal induction of *C. albicans* (10^6^ cells/ml) during co-incubation with (**a**) live *L. rhamnosus* GG cells, cell-free supernatant (CFS), UV-inactivated cells and heat-killed cells (10^8^ cells/ml); (**b**) the isolated lectin-like proteins Llp1 and Llp2 (50 µg/mL) and purified EPS (200 µg/mL) from *L. rhamnosus* GG; (**c**) different concentrations of Msp1 from *L. rhamnosus* GG; (**d**) different concentrations of lactic acid (50% L-lactic acid and 50% D-lactic acid) and (**e**) the combination of lactic acid (mixed, D-lactic acid and L-lactic acid) and Msp1; (**f**) Biofilm formation during co-incubation with *L. rhamnosus* GG cell-free supernatant (CFS) (20%), lactic acid (0, 4%) and Msp1 (10 µg/mL), with MRS broth and water as respective controls. The results were normalized to hyphal formation and biofilm formation of *C. albicans* solely. Single and double asterisks indicate respectively p-values below 0.1 and 0.01, compared to *C. albicans* solely.

To further elucidate how *Lactobacillus* can impact hyphal morphogenesis, we first explored whether the contributing *L. (para)casei/rhamnosus* factors are surface-bound, secreted, or both. *L. rhamnosus* GG was chosen as model, since this strain is well-characterized at genetic and molecular level^42^. We first compared the effect of live *L. rhamnosus* GG cells on serum-induced hyphal formation to its cell-free culture supernatant, containing solely secreted molecules, and to UV-inactivated or heat-killed *L. rhamnosus* GG cells. Cells treated in both ways should no longer secrete molecules, but in contrast to the heat-killed cells, the surface proteins of the UV-inactivated cells should not be denatured. We showed that the supernatant from *L. rhamnosus* GG inhibited hyphal formation almost completely (97%), whereas the UV-inactivated *L. rhamnosus* GG cells inhibited hyphal formation of *C. albicans* to the same extent as live cells (57% and 51%, respectively) (Fig. 2a). The heat-killed cells, on the other hand, were no longer able to inhibit *C. albicans* hyphal formation. These results thus indicate that the main core *L. rhamnosus*-specific effectors molecules are secreted, but can also be surface-bound or are supplemented by a heat-sensitive cell-bound effector.

Next, we explored the activity of the major documented *L. rhamnosus* GG surface molecules that could have putative hyphae-binding properties due to lectin-sugar interactions. Key candidates for hyphae-binding include the lectin-like protein 1 (Llp1) and 2 (Llp2)^43^, the galactose-rich exopolysaccharides (EPS)^44^ and the major secreted protein 1 (Msp1), which is mannosylated^45^.

Llp1 and Llp2 have been shown to bind to D-mannose and the complex sugar mannan by sepharose-binding and glycan array screening^43^, both of which are present in the outer layer of *C. albicans* cell wall^3,6^. We therefore aimed to explore whether this sugar-binding capacity could also result in interference with hyphal morphogenesis. Treatment with Llp1 and Llp2 did not, however, show a reduction of *Candida* hyphal formation at 50 µg/ml (Fig. 2b), a previously documented active antibacterial concentration^43^. Proteins with lectin-like properties can also be found on the hyphal surface^46,47^, rendering the glycoconjugates on the lactobacillary surface potential interaction partners as well. In agreement with previous results^48^, isolated EPS from *L. rhamnosus* GG was able to inhibit hyphal morphogenesis, but only at a rather high concentration of 200 µg/mL (Fig. 2b). In contrast, the peptidoglycan hydrolase Msp1 from *L. rhamnosus* GG tested here demonstrated a remarkably strong inhibitory activity (Fig. 2c), reducing hyphal morphogenesis with more than 50%, at concentrations as low as 5 µg/mL. To check whether Msp1 was only inhibiting hyphal morphogenesis and not the viability of *C. albicans*, we determined the growth capacity of the *C. albicans* cells after three hours and six hours of hyphal induction in presence of Msp1. This showed that the viability of the *C. albicans* was not affected during the treatment with Msp1 (Supplementary Fig. S1).

Although the production of lactic acid by the lactobacilli could not really explain the observed variation in anti-hyphal activity between different *Lactobacillus* strains (Fig. 1), we also exogenously added lactic acid in this screening to quantify its contribution to the antihyphal activity of *L. rhamnosus* GG. Lactic acid as such, at naturally occurring culture supernatant concentrations (1%, a combination of D- and L-lactic acid in a 1:1 ratio), also reduced morphogenesis by approximately 50% (Fig. 2d).

Since the supernatant showed very strong activity and since Msp1 and lactic acid are major components of the supernatant, we next investigated whether Msp1 could act synergistically with lactic acid. The combination of lactic acid at a lower concentration than present in the supernatant (0.5%) and Msp1 (2 µg/ml) was shown to decrease hyphal formation more than 94%, a level of inhibition comparable to the cell-free supernatant, indicating this combination contained the main effectors conferring the anti-hyphal activity to *L. rhamnosus* GG (Fig. 2e). Since we observed a negative correlation between D-lactic acid production and hyphal inhibition and since the best-performing strains mainly produced L-lactic acid, we compared the synergistic effect of mixed lactic acid on Msp1 activity to both isomers separately. Remarkably, this comparison showed no differences between the isomers (Fig. 2e).

Hyphal morphogenesis is tightly linked to biofilm regulation of *C. albicans*^49^, we therefore next investigated whether *L. rhamnosus* GG could also inhibit *C. albicans* biofilm formation. This experimental set-up revealed that the supernatant of *L. rhamnosus* GG was able to decrease biofilm formation of *C. albicans*. The two main components of the supernatant, lactic acid and Msp1, separately also showed anti-biofilm activity, however no clear synergistic effect was observed with the concentrations of lactic acid and Msp1 tested (Fig. 2f).

### Mutant analysis of *L. rhamnosus* GG supports key role for Msp1

As mutual interactions between the individual molecules on the lactobacillary surface might strengthen or attenuate the anti-hyphal activity of individual purified molecules, we performed additional experiments with specific *L. rhamnosus* GG isogenic mutants available from our previous research (see Materials and methods section)^42^. This complementary approach also allowed us to study molecules that could not be purified to a sufficient level.

**Figure 3:**
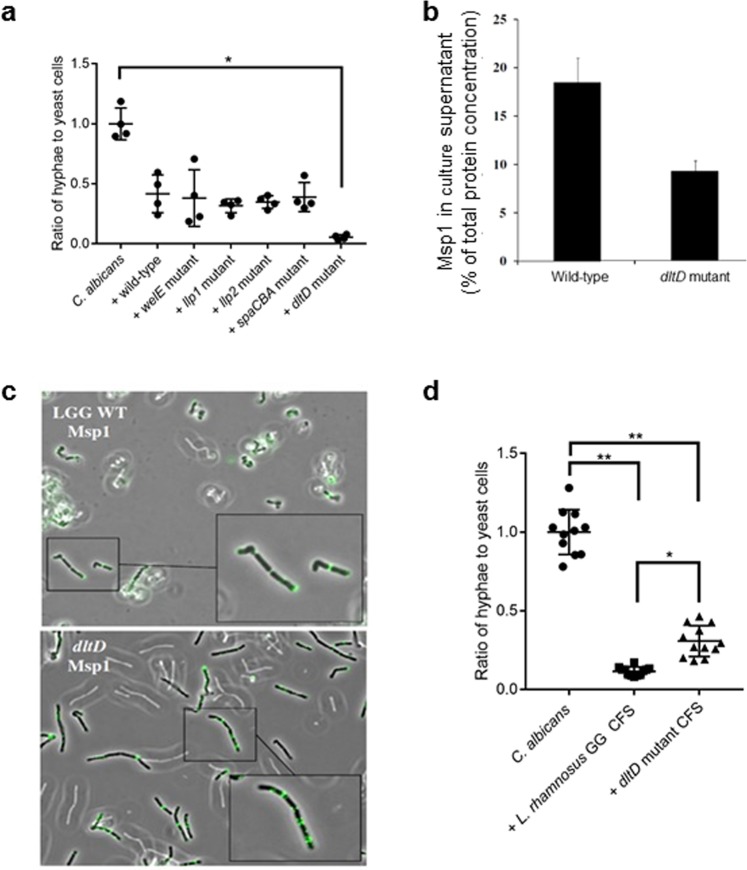
Mutant analysis supports key role for Msp1 in anti-hyphal activity. (**a**) Hyphal induction of *C. albicans* (10^6^ cells/ml) during co-incubation with *L. rhamnosus* GG mutant strains, lacking long galactose-rich EPS, Llp1, Llp2, SpaCBA pili or D-alanylation of the lipoteichoic acids (LTA) on their surface. The hyphal inhibition levels were normalized to inhibition level of *L. rhamnosus* GG wild-type. An asterisk indicate p-values below 0.001, compared to *L. rhamnosus* GG wild-type. (**b**) Visualization of Msp1 on the surface of wild-type (upper panel) and *dltD* mutant cells (lower panel) by indirect immunofluorescence using light microscopy. (**c**) Quantification of proteins in culture supernatant of *L. rhamnosus* GG WT and the *dltD* mutant using ELISA. (**d**) Hyphal induction of *C. albicans* (10^6^ CFU/ml) during co-incubation with cell-free supernatant from *L. rhamnosus* GG wild-type and from its *dltD* mutant strain. Single and double asterisks indicate respectively p-values below 0.001 and 0.0001, compared to *C. albicans* solely.

Mutant analysis confirmed that the presence or absence of the EPS layer and lectins does not play a crucial role in the anti-hyphal activity of *L. rhamnosus* GG cells, as shown in Fig. 3a. Previous research showed the importance of the SpaCBA pili and their fucose and mannose residues in *L. rhamnosus* GG interactions with host cells and glycoconjugates, such as intestinal mucus^50,51^, of which structural homologs might be present on the hyphal surface. These complex, heteropolymeric SpaCBA pili themselves are difficult to purify^50–52^, therefore we included the isogenic *spaCBA* mutant of *L. rhamnosus* GG in the mutant analysis. This showed that the presence or absence of these SpaCBA pili did not play a significant role in the anti-hyphal activity of *L. rhamnosus* GG (Fig. 3a).

Due to the central role of Msp1 in bacterial growth and cell separation, an isogenic knock-out mutant is not available in *L. rhamnosus* GG^53^. However, the *dltD* mutant is an interesting generic surface mutant of *L. rhamnosus* GG, because the lipoteichoic acids are no longer D-alanylated, resulting in dramatic shifts in surface charge and association with surface proteins and other molecules^54^. Remarkably, the hyphal morphogenesis of *C. albicans* was almost completely abolished by *L. rhamnosus* GG *dltD* mutant cells. To explore whether this could also be explained by the activity of Msp1, we checked whether Msp1 stayed more associated with the surface of *dltD* mutant cells after secretion than in the wild-type cells. Fluorescently labelled anti-Msp1 antibodies showed that Msp1 was indeed a twofold less secreted in the supernatant of the *dltD* mutant (Fig. 3b) and appeared to be present in higher concentration on the surface of these mutant cells (Fig. 3c). This thus probably resulted in a higher bio-availability of Msp1 in experiments using the *dltD* mutant cells as compared to wild-type cells. The consequential comparison between the effects of the supernatant from *L. rhamnosus* GG wild-type and *dltD* mutant on hyphal morphogenesis showed that the lower secretion of Msp1 in the supernatant of the *dltD* mutant indeed resulted in a significantly lower inhibition (p = 0.0001) (Fig. 3d).

The combination of the approach using either mutants or isolated molecules thus further demonstrated the key role for Msp1 in the anti-hyphal activity of *L. rhamnosus* GG. This finding is in agreement with the fact that the other tested strains from the *L. casei* group showed strong activity (Fig. 1a), since Msp1 has been shown to be conserved among - at least a part of - the *L. casei* group^45^, while the other studied molecules are rather specific for the strain *L. rhamnosus* GG.

### Msp1 shows chitinase activity, independent of its glycosylation state

**Figure 4:**
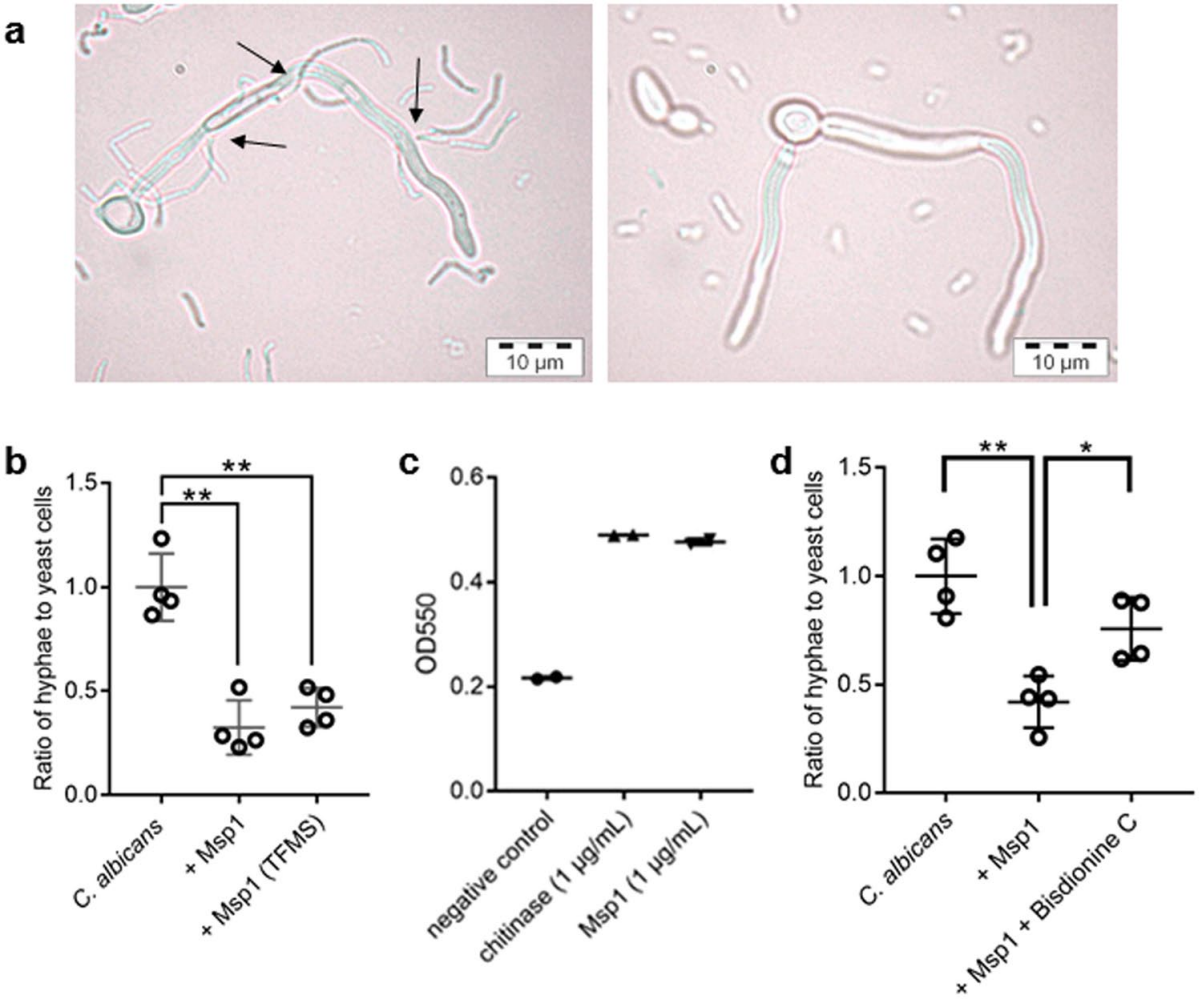
Enzymatic activity of Msp1. (**a**) Microscopic images of *L. rhamnosus* GG (left) and *L. plantarum* WCFS1 (right) after incubation with *C. albicans* hyphae. Arrows indicate sites where the poles of lactobacilli seem to interact with the hyphae. Representative images are shown. (**b**) Chemical deglycosylation of Msp1 does not influence its anti-hyphal activity. (**c**) Msp1 can break down chitin-azure, a chitin derivative. As a control, the sodium acetate buffer was used. (**d**) The chitinase inhibitor, Bisdionine C, can prevent hyphal inhibition partially. The results on hyphal inhibition were normalized to hyphal formation of *C. albicans* solely. Asterisks indicate p-values below 0.05, double asterisks indicate p-values below 0.01, compared to *C. albicans* solely.

We subsequently aimed to explore the interaction between Msp1 and *Candida* cells in more detail. First, we compared the binding to hyphal cells between *L. rhamnosus* GG, as a strong anti-hyphal strain, and *L. plantarum* WCFS1, being one of the least effective strains tested previously (Fig. 1a). These strains belong to the limited number of *Lactobacillus* strains whose main peptidoglycan hydrolases have been thoroughly characterized^53,55^. Both their major peptidoglycan hydrolases have been shown to be localized at the poles of the *Lactobacillus* cells, but they differ in hydrolytic activity and glycosylation state: Msp1 has documented γ-D-glutamyl-L-lysyl-endopeptidase activity^53^ and appears to be glycosylated with mannose residues^45^, while Acm2 from *L. plantarum* WCFS1 was identified as an endo-β-acetylglucosaminidase^55^ and appears to glycosylated with *N*-acetylglucosamine residues^56^. We first explored whether these dissimilarities are reflected in a different interaction of the *Lactobacillus* strains with the hyphae. Microscopic inspection of *C. albicans* hyphae after induction in presence of *L. rhamnosus* GG revealed that the *Lactobacillus* poles appeared to be the main contact point with the hyphal cells (Fig. 4a, right panel). In contrast to *L. rhamnosus* GG, *L. plantarum* WCFS1 cells did not appear to closely interact with the hyphae (Fig. 4a, left panel), suggesting that the close binding of *L. rhamnosus* GG poles to the hyphae is important for its anti-hyphal activity. Counting the attached and unattached *Lactobacillus* cells in three different repeats showed that 60 ± 6% of the *L. rhamnosus* GG bound to the hyphae, while none of *L. plantarum* WCFS1 did (data not shown).

To explore whether the binding between Msp1 and *C. albicans* hyphae could indeed be due to their sugar-lectin interactions, as suggested above, we next investigated the activity of non-glycosylated Msp1. After chemical deglycosylation, the level of hyphal inhibition showed to be similar to native (glycosylated) Msp1 (Fig. 4b), indicating that another mechanism probably underlies the anti-hyphal activity of Msp1.

Despite their different origin, chitin from *C. albicans* and peptidoglycan from *L. rhamnosus* GG show some structural similarities due to the presence of *N*-acetylglucosamine residues in both their backbones. Because of this, and because of the close contact between the *Lactobacillus* poles and the hyphae, we hypothesized that Msp1 might be able to use chitin, the main polymer of the hyphal cell wall, as a substrate. Based on assays with chitin-azure, we found that Msp1 is indeed able to break down chitin, to the same extent as a commercially available chitinase from *Streptomyces griseus* (Fig. 4c). Finally, we determined whether a chitinase inhibitor would be able to restore *C. albicans* hyphal morphogenesis. Bisdionine C, a known chitinase inhibitor, partially reversed the inhibitory effects of Msp1 on hyphal morphogenesis (Fig. 4d), further substantiating the chitinase activity as basis for the anti-hyphal capacity of Msp1.

## Discussion

In the present study, we showed that certain *Lactobacillus* taxa can inhibit hyphal morphogenesis of *C. albicans* more efficiently than others. More specifically, we demonstrated that the major secreted protein and main peptidoglycan hydrolase of *L. rhamnosus* GG, Msp1, is the key effector and can reduce hyphal formation by its chitinase activity, especially in combination with lactic acid, another important metabolite of lactobacilli.

Our findings on the complete inhibition of hyphal formation by the supernatant from *L. rhamnosus* GG is in line with previous observations on the effect of *L. rhamnosus* LR32 supernatant on hyphae density in *C. albicans* biofilms^35^. Moreover, a comparison between live cells and both UV-inactivated and heat-killed cells provided novel insights into the underlying molecular mechanism, as the effectors need to be structurally intact, but not necessarily actively secreted during the hyphal induction.

**Figure 5:**
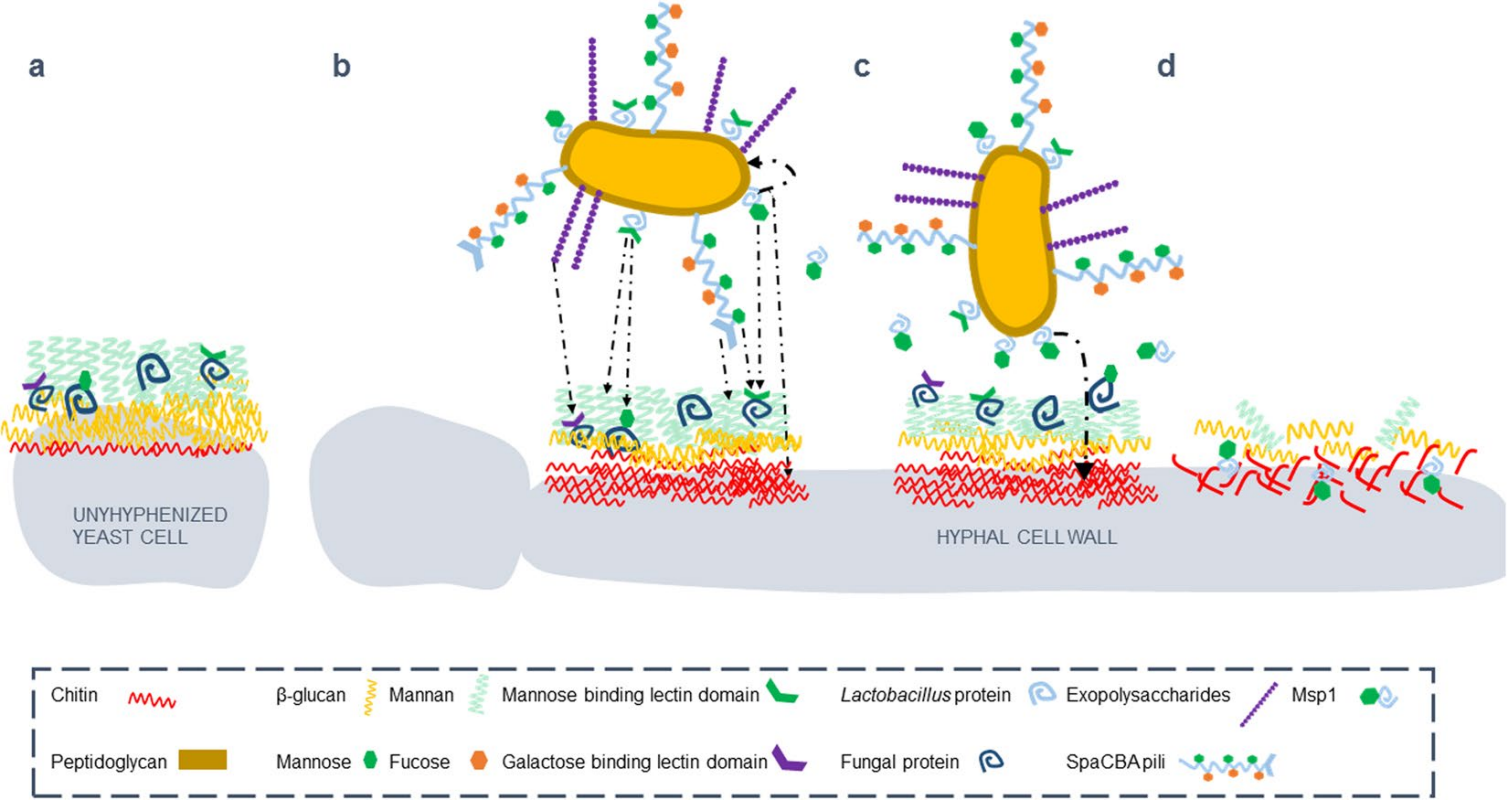
A schematic representation of the proposed mode of action underlying the anti-*C. albicans* activity of *L. rhamnosus* GG and the other possible, investigated targets. On the surface of *L. rhamnosus* GG, several potential interaction partners for components on the cell wall of *C. albicans* cells can be found. The potential interactions between *C. albicans* and *L. rhamnosus* GG surface components that were tested in this manuscript are indicated with black arrows. In the hyphal cell wall, the proportion of chitin is much higher (**b**) than in unhyphenised cells (**a**), which makes the polymer available for the hydrolytic activity of Msp1 (**c**). Subsequent contact with Msp1 causes degrading and destabilizing the hyphal cell wall (**d**). The size proportions between *C. albicans* and the lactobacilli are not respected for clarity.

The multilayered cell wall of *C. albicans*, existing of an inner layer of chitin and β-glucans and an outer layer of mannans and (glycosylated) proteins^6^, offers several potential target sites for the binding with lactobacillary factors. We tested different secreted and surface-bound molecules from *L. rhamnosus* GG, which are represented in the schematic overview in Fig. 5. By combining the results on anti-hyphal activity of purified molecules with these of different mutant strains, we found the combination of Msp1 and lactic acid to be the key effectors and synergistically abolish hyphal morphogenesis.

The degrading effects of chitinases on a yeast cell wall were first described for the fungus *Trichoderma viride*^57^, but they have to the best of our knowledge not yet been described for bacterial peptidoglycan hydrolases nor for any lactobacillary protein. Although chitin and peptidoglycan share some structural similarities, namely the presence of N-acetylglucosamine residues in their backbones, the previously studied peptidoglycan hydrolase activity of Msp1 was not shown to involve these bounds. Msp1 was shown to carry γ-D-glutamyl-L-lysyl-endopeptidase activity^53^, cutting between the D-glutamine and L-lysine residues in the peptide stem in bacterial peptidoglycan. This peptidoglycan hydrolase activity was also shown to be conserved among a number of the tested *L. casei* group strains^45^ and was also found in *L. casei* BL23^58^. Unfortunately, structural information on peptidoglycan composition and accompanying hydrolase activity in *Lactobacillus* is quite limited. In *L. plantarum* WCFS1, the hydrolase responsible for cell septation (Acm2) was identified as an endo-β-acetylglucosaminidase^55^, and in *L. gasseri* DSM 20243, the major peptidoglycan hydrolase was shown to have N-acetylmuramidase activity^59^. Microscopic examination and anti-hyphal experiments with *L. plantarum* WCFS1 indicate that Msp1 does not share its chitinase activity with other types of peptidoglycan hydrolases. In *L. fermentum*, however, D-glutamine and lysine were also observed in the peptide stem^60^. This indicates that *L. fermentum* strains might have a similar peptidoglycan structure and possibly a similar main peptidoglycan hydrolase activity as *L. rhamnosus* GG, which could explain that the inhibition level of *L. fermentum* AMBV1 was almost to the same extent as some *L. casei* strains. Yet, the exact enzymatic activity remains to be substantiated in follow-up studies.

In light of the observed chitinase activity of Msp1, a number of factors could explain the synergistic effects with lactic acid. Firstly, Msp1, as a hydrolase, has an acidic pH optimum^53^. Secondly, while *C. albicans* is known for its acid tolerance, the proportion of chitin in the hyphal cell wall has been shown to be even more increased in an acid environment^39^. Thirdly, although we could not observe a difference in synergistic effect with Msp1 between D- and L-lactic acid at the tested concentrations, the presence of L-lactic acid, the main isomer in the supernatant of *L. casei* group strains, has been shown to result in glucan masking on the hyphal surface^40^, potentially making the hyphae more sensitive/accessible to the chitinase activity of Msp1.

The chitinase activity of Msp1 might also be relevant for non-hyphae producing *Candida* species, as it has been described that the chitin levels are elevated in *C. glabrata* during infections in a murine colitis model^61^. In addition, in Crohn’s disease patients, both an increase in *C. glabrata* and a decrease in *Firmicutes* was found to characterize the gut microbiota^62^.

Lactobacilli and their specific properties are often evaluated at strain-level. Thanks to the approach in this study, we could suggest that this *C. albicans* hyphae inhibitory activity is possibly present in most strains belonging to the *L. casei* group due to their specific peptidoglycan structure and accompanying hydrolases. In this way, the study gives additional indications that probiotic mechanistic research should not only be performed on strain-level to find core properties^36^. The effects of *Lactobacillus* strains expressing this specific type of peptidoglycan hydrolase and whether they show stronger potential as anti-*C. albicans* strategy than others, should of course still be substantiated with *in vivo* evidence. Although it is difficult to explain the mixed results of clinical trials based on our findings in hindsight, since often the applied *Lactobacillus* strains are not specified to strain- or species level or a probiotic mixture and different formulations were often used, *L. casei* group strains were used in a number of clinical trials with positive outcomes. Treatment with *L. rhamnosus* GR-1 (formulated in gelatin capsules) improved symptoms in women suffering from vulvovaginal candidosis^15^ and *L. rhamnosus* HS111 (formulated as dry powder in capsules) contributed to a significant reduction of *Candida* infection in the oral cavity^63^. In contrast, a clinical trial assessing *L. casei* Shirota on *Candida* with negative results actually investigated *Candida* viability rather than virulence^64^, which would not be affected by the hyphal-inhibitory activity of the lactobacilli. Of course, when evaluating a *Lactobacillus* strain or species for its anti-*Candida* potential, other factors than inhibition of hyphae should be considered. For example, *L. plantarum* CMPG5300 did not show high hyphal formation inhibition rates (30%) but has previously been shown to co-aggregate with *C. albicans* and may in this way inhibit *C. albicans* adhesion and contribute to disease prevention^65^. Depending on the niche, other factors may play a role for applying lactobacilli as an anti-*Candida* therapy, such as the epithelial adhesion of *L. rhamnosus* GG to the gastro-intestinal tract by its SpaCBA pili^51^ and *L. rhamnosus* GR-1 to the vaginal mucosa by its Llp1 lectin^66^. Additional aspects of clinical trials will also influence the outcome, such as the production or formulation, including encapsulation, of the probiotics^67^ and organisation of clinical trials, including randomisation and the inclusion of control groups^68,69^.

In conclusion, our data demonstrate that selected *Lactobacillus* taxa show stronger *C. albicans* hyphae inhibition activity than others, especially the taxa belonging to the *L. casei* group. These taxa appear to owe this inhibitory activity to their major peptidoglycan hydrolase, breaking down the main polymer of the hyphal cell wall, chitin. The identification of the peptidoglycan hydrolase as a core probiotic property helps to unravel the complex interactions between probiotic bacteria and *Candida* species, and can assist in the selection of proper probiotic strains for use as potential probiotics in patients with *Candida* infections or at risk for frequent recurrences of it.

## Materials and Methods

### Microbial strains and culture conditions

*Lactobacillus* strains (Table 1) were grown at 37 °C without agitation in de Man, Rogosa and Sharpe (MRS) broth (Difco, Erembodegem, Belgium). *C. albicans* SC5314 was grown in yeast extract peptone dextrose (YPD) broth (Carl Roth, Karlsruhe, Germany) at 37 °C and with continuous shaking^70^.

**Table 1:** Bacterial strains used in this study.

Strain	Reference	Description	Characteristics
*L. rhamnosus* GG ATCC 53103	ATCC^76^	Wild-type	Intestinal isolate
*L. rhamnosus* CMPG5351	^77^	*welE* mutant of *L. rhamnosus* GG	Lacks long, galactose-rich exopolysaccharides and shows increased exposure of SpaCBA pili
*L. rhamnosus* CMPG5540	^54^	*dltD* mutant of *L. rhamnosus* GG	Lacks D-alanylation of lipoteichoic acid and increased exposure of certain surface proteins
*L. rhamnosus* CMPG5357	^51^	*spaCBA* mutant of *L. rhamnosus* GG	Lacks expression of spaCBA pili
*L. rhamnosus* CMPG10701	^43^	*llp1* mutant of *L. rhamnosus* GG	Lacks expression of Llp1 lectin
*L. rhamnosus* CMPG10706	^43^	*llp2* mutant of *L. rhamnosus* GG	Lacks expression of Llp2 lectin
*L. rhamnosus* GR-1 ATCC 5582	ATCC^78^	Wild-type	
*L. casei* AMBR2	^41^	Wild-type	
*L. casei* ATCC 393	ATCC^79^	Wild-type	
*L. paracasei* ATCC 334	ATCC^79^	Wild-type	
*L. pentosus* KCA1	^80^	Wild-type	
*L. pentosus* ATCC 8041	ATCC^81^	Wild-type	
*L. plantarum* WCFS1	ATCC^82^	Wild-type	
*L. plantarum* CMPG5300	^83^	Wild-type	
*L. reuteri* RC-14 ATCC 55845	ATCC^78^	Wild-type	
*L. reuteri* AMBV38	In-house	Wild-type	Vaginal isolate
*L. fermentum* AMBV1	In-house	Wild-type	Vaginal isolate
*L. gasseri* AMBV2	In-house	Wild-type	Vaginal isolate
*L. gasseri* AMBV10	In-house	Wild-type	Vaginal isolate
*L. gasseri* AMBV28	In-house	Wild-type	Vaginal isolate
*L. gasseri* AMBV47	In-house	Wild-type	Vaginal isolate
*L. jensenii* AMBV103	In-house	Wild-type	Vaginal isolate
*L. crispatus* LMG12004	BCCM^84^	Wild-type	
*L. crispatus* AMBV6	In-house	Wild-type	Vaginal isolate
*L. crispatus* AMBV104	In-house	Wild-type	Vaginal isolate

The in-house *Lactobacillus* isolates were taxonomically characterized to the species level by sequencing the 16S ribosomal RNA gene. Briefly, the complete *16S rRNA* gene (1.5 kb) was amplified with the universal 27 F and 1492 R primers and sequenced. The obtained sequences were compared with reference 16S rRNA gene sequences by BLAST analysis at the National Center for Biotechnology Information (NCBI) website (https://blast.ncbi.nlm.nih.gov/Blast.cgi).

The in-house *Lactobacillus* isolates were collected during a clinical study (Nr 20040719) that was reviewed and approved by the ethical committee of regional hospital of Tienen (Belgium) and all patients gave their explicit consent before sampling.

### Inhibition of hyphal formation in *C. albicans*

Hyphal growth of *C. albicans* was induced by supplementing YPD broth with 10% heat inactivated fetal bovine serum (FBS) (Thermo Fischer, Asse, Belgium), while incubated with or without lactobacilli (10^8^ CFU/ml) or purified molecules. After 3 hours of incubation, at least a hundred yeast cells and/or hyphae in four biological repeats were counted microscopically and the ratio of hyphae to yeast cells was calculated.

### Viability of *C. albicans*

The viability of *C. albicans* during hyphae formation and hyphae-inhibitory treatments was checked by quantifying the viable plate count at 3 and 6 hours of incubation with the macrodilution method on YPD agar.

### Inhibition of *C. albicans* biofilm development

The inhibiting effects on *C. albicans* biofilms were assessed as described previously by^71^. Briefly, 8 × 10^4^ *C. albicans* cells were added to the wells of a 96 well plate, together with the samples (supernatant, lactic acid, Msp1) or controls (MRS or H_2_O). After incubation for 24 h at 37 °C, the biofilms were washed twice and then 2,3-bis(2-methoxy-4-nitro-5-sulfophenyl)-2*H*-tetrazolium-5-carboxanilide (90 µl, 1 mg/ml) (Sigma Aldrich) and phenazine methosulphate (10 µl, 0.2 mg/ml) (Sigma Aldrich) were added to the wells. After a second incubation (37 °C, 30 minutes, in the dark), the absorbance at 492 nm was measured using a Synergy HTX multi-mode reader (Biotek, Drogenbos, Belgium).

### UV-inactivation and heat-killing of lactobacilli

After two washing steps, lactobacilli were UV-inactivated by three repeats of 15 minutes of UV irradiation, and heat-killed by incubating 20 minutes at 80 °C. Inactivation was confirmed by plating on MRS agar.

### Preparation of cell-free supernatant

Overnight cultures of lactobacilli were grown without agitation in MRS medium at 37 °C. Cell-free supernatant was prepared by centrifuging the culture at 2000 × g for 10 min at 4 °C and then filtering through 0.2 µm filters (VWR, Haasrode, Belgium).

### D- and L-lactic acid production

After overnight incubation, cell-free supernatant was obtained by centrifugation (10 min, 2000 × g, 4 °C) and filter sterilization through 0.2 µm filters. The concentration of D- and L-lactic acid was measured with the commercially available kit from R-Biopharm (Darmstadt, Germany).

### Isolation of Llp1 and Llp2 from *L. rhamnosus* GG

The Llp1 and Llp2 proteins from *L. rhamnosus* GG were isolated as described before^43^. Briefly, the production of the recombinant protein was induced with 1 mM isopropyl β-D-thiogalactopyranoside (IPTG) in recombinant *E. coli* BL21 cells expressing the lectins (CMPG10708 and CMPG10709). After incubation (25 °C, shaking), the pellets were suspended in non-denaturing lysis buffer (50 mM NaH_2_PO_4_, 300 mM NaCl and 20 mM imidazole) and sonicated to release the soluble recombinant lectins from the cells. Afterwards, the lectins were purified using affinity chromatography with a HisTrap^™^ HP column (GE Healthcare) and size exclusion chromatography with a Highload^™^ 16/60 column packed with a matrix of Superdex^™^ prep grade (GE Healthcare).

### Isolation of Msp1 from *L. rhamnosus* GG

Msp1 was purified by cationic exchange chromatography as described previously^45,72^. Briefly, the culture supernatant was loaded onto SP Sepharose High Performance (GE Healthcare), equilibrated with 60 mM lactate buffer (pH 4.0). Lactate buffer containing ascending NaCl concentrations (100–1000 mM) was used to elute bound protein. Fractions containing Msp1 were identified using SDS-PAGE and concentrated using Vivaspin filters (MW cut off 10,000) (Sartorius Stedim biotech GmbH, 37070 Goettingen, Germany).

### Deglycosylation of Msp1

Msp1 was chemically deglycosylated by trifluoromethanesulphonic acid (TFMS) method (−20 °C, for 30 minutes), as described before^73^. After treatment, the proteins were extensively dialyzed and analyzed by SDS-PAGE.

### Chitinase activity of Msp1

First, the chitinase activity of Msp1 was investigated based on breakdown of chitin-azure (Sigma), as described previously^74^. Further confirmation was based on inhibition of chitinase activity by 2.5 mM Bisdionine C (Sigma), as described previously^75^.

### Indirect immunofluorescence using light microscopy

Anti-Msp1 rabbit antiserums were used on wild-type and *dltD* mutant cells. Anti-rabbit IgG antibodies conjugated with Alexa Fluor 488 were used to visualize Msp1 localization on the cells. Samples were visualized with a Zeiss Axio Imager Z1, equipped with an AxioCam MRm Rev.3 monochrome digital camera. The samples were imaged with a ‘Plan-Neofluar’ 100x/1.3 Oil Ph3 objective. Images were analysed with the supplied AxioVision Rel.4.6 software making overlays of phase-contrast and fluorescent images.

### ELISA

The protein concentration in the cell-free supernatant of *L. rhamnosus* GG and CMPG5540 was determined using bicinchoninic acid (BCA) protein assay. The wells of a 96- well ELISA plate (Greiner, Bio-one) were coated overnight with supernatant (0.5 µg/mL), after lyophilization and resolving in PBS, or Msp1 (at different concentration, standard curve) at 37 °C. Afterwards, the wells were washed three times with PBS/T (PBS with 0.05% Tween 20), 250 µL PBS/T with 25% solution of skimmed milk was added, followed by a 1 hour incubation at 37 °C to block aspecific binding. Next, the wells were washed three times with PBS/T and each well was then filled with 100 µL of Msp1 antiserum diluted 1:2000 in PBS/T and incubated (37 °C, 90 min). Alkaline phosphate-conjugated goat anti-rabbit immunoglobulin G (IgG, Sigma) was diluted 1:3000 in PBS/T and added to each well (100 µL) before incubation (37 °C, 1 h). After incubation (30 min, 37 °C) of the bound antibodies with 150 µL of p-nitrophenyl phosphate (1 mg/mL in 1 M Tris-HCl, pH 9.8) (Sigma) per well, the absorbance (405 nm) of each well was read with a Synergy MX microtiter plate reader (Biotek Instruments).

### Statistics

Shapiro-Wilk normality test (GraphPad Prism 7.02, CA, USA) was used to determine whether the data are normally distributed. Statistical significance between conditions was estimated by one-way ANOVA and Tukey’s multiple comparisons test.

## Supplementary information

Fig S1
