# Supplementary material for: Inhibition of Candida albicans morphogenesis by chitinase from Lactobacillus rhamnosus GG
Source: Sci Rep. 2019 Feb 27;9:2900. doi: 10.1038/s41598-019-39625-0 (PMC6393446; doi:10.1038/s41598-019-39625-0)
Supplement: Supplementary file 1 — Fig S1 [file 41598_2019_39625_MOESM1_ESM.pdf]

Inhibition of *Candida albicans* morphogenesis by chitinase from *Lactobacillus rhamnosus* GG

Camille Nina Allonsius[a], Dieter Vandenheuvel[a], Eline F. M. Oerlemans[a], Mariya I. Petrova[a], Gilbert G. G. Donders[b,c], Paul Cos[d], Peter Delputte[d], Sarah Lebeer[a,*]

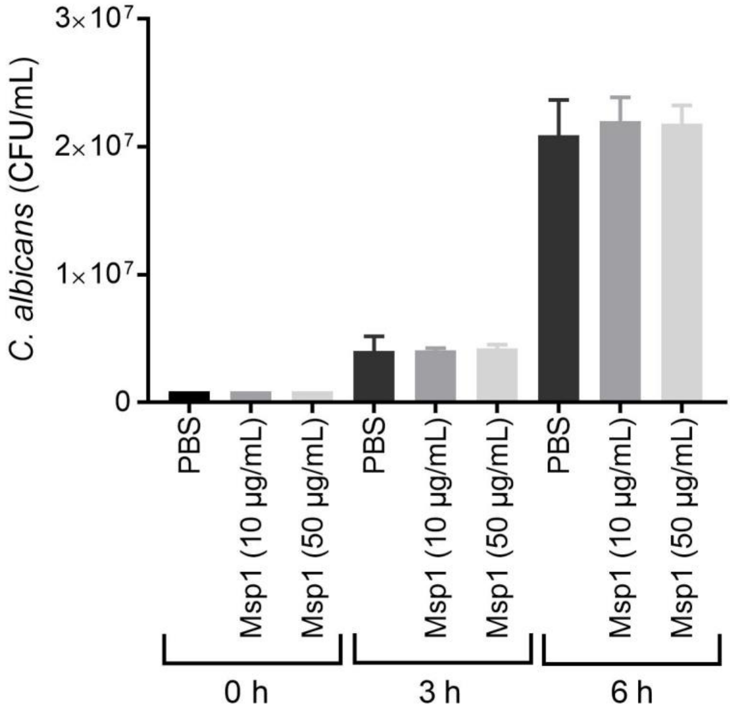

Supplementary Fig S1. Viability of *C. albicans* during hyphal morphogenesis in presence of Msp1. Density of *C. albicans* (starting from 10^6 cells/ml) after 3 hours and 6 hours of hyphal induction during co-incubation with Msp1 from *L. rhamnosus* GG (10 and 50 µg/mL). As a negative control, PBS was used.
